# Through the client’s eyes: using narratives to explore experiences of care transfers during pregnancy, childbirth, and the neonatal period

**DOI:** 10.1186/s12884-017-1369-6

**Published:** 2017-06-12

**Authors:** Cherelle M.V. van Stenus, Mark Gotink, Magda M. Boere-Boonekamp, Anneke Sools, Ariana Need

**Affiliations:** 1 Institute for Innovation and Governance Studies, Departments of Public Administration, and Health Technology and Services Research, University of Twente, P.O. Box 217, 7500 AE Enschede, The Netherlands; 2 Department of Youth Healthcare, GGD Twente, P.O. Box 1400, 7500 BK Enschede, The Netherlands; 3 Institute for Innovation and Governance Studies, Department of Health Technology and Services Research, University of Twente, P.O. Box 217, 7500 AE Enschede, The Netherlands; 4 Institute for Innovation and Governance Studies. Department of Psychology, Health and Technology, University of Twente, P.O. Box 217, 7500 AE Enschede, The Netherlands; 5 Institute for Innovation and Governance Studies, Department of Public Administration, University of Twente, P.O. Box 217, 7500 AE, Enschede, The Netherlands

**Keywords:** Client experiences, Pregnancy, Transfers of care, Narratives, Quality aspects, Perinatal healthcare

## Abstract

**Background:**

The client experience is an important outcome in the evaluation and development of perinatal healthcare. But because clients meet different professionals, measuring such experiences poses a challenge. This is especially the case in the Netherlands, where pregnant women are often transferred between professionals due to the nation’s approach to risk selection. This paper explores questions around how clients experience transfers of care during pregnancy, childbirth, and the neonatal period, as well as how these experiences compare to the established quality of care aspects the Dutch Patient Federation developed.

**Method:**

Narratives from 17 Dutch women who had given birth about their experiences with transfers were collected in the Netherlands. The narratives, for which informed consent was obtained, were collected on paper and online. Storyline analysis was used to identify story types. Story types portray patterns that indicate how clients experience transfers between healthcare providers. A comparative analysis was performed to identify differences and similarities between existing quality criteria and those clients mentioned.

**Results:**

Four story types were identified: 1) Disconnected transfers of care lead to uncertainties; 2) Seamless transfers of care due to proper collaboration lead to positive experiences; 3) Transfers of care lead to disruption of patient-provider connectedness; 4) Transfer of care is initiated by the client to make pregnancy and childbirth dreams come true. Most of the quality aspects derived from these story types were identified as being similar or complementary to the Dutch Patient Federation list. A ‘new’ aspect identified in the clients’ stories was the influencing role of prior experiences with transfers of care on current expectations, fears, and wishes.

**Conclusions:**

Transfers of care affect clients greatly and influence their experiences. Good communication, seamless transfers, and maintaining autonomy contribute to more positive experiences. The stories also show that previous experiences influence client’s expectations for the next pregnancy, childbirth, and transfers of care.

**Electronic supplementary material:**

The online version of this article (doi:10.1186/s12884-017-1369-6) contains supplementary material, which is available to authorized users.

## Background

Quality of care is considered a multidimensional concept, illustrated by an extensive body of literature that emerged over the last four decades [1–7]. In addition to general assessment criteria for quality of care, criteria have been developed for specific types of care, such as the quality of perinatal healthcare. The quality of the perinatal healthcare system is largely assessed with objective measures of health outcomes, such as mortality or morbidity, caesarean section, and premature birth rates [8]. Clients’ experiences (primarily the women involved) have become an important outcome in the evaluation and development of perinatal healthcare services [9].

It is argued that demand-driven healthcare and improvements in the quality of care are not possible without clients’ input [10, 11]. Documenting experiences provides an important source of information for healthcare professionals, managers, and policymakers that can be directed towards improving the quality of care and experiences [12].

Looking at perinatal healthcare in particular, the Dutch Patient Consumers Federation NPCF (Nederlandse Patienten Consumenten Federatie) developed a list of quality aspects healthcare in 2014, based on clients’ experiences of the perinatal healthcare system in general [13]. The aim of the NPCF was to identify the wishes and needs of clients with respect to the entire perinatal period, and to clarify what qualitatively good healthcare entails for these women. This has resulted in a list of ten quality aspects that clients considered most important [13] (Table 1).

**Table 1:** Quality aspects of perinatal healthcare described by the Dutch Patient Consumers Federation (NPCF)^a^

Autonomy	The client is able to make choices regarding guidance and provision of care. The professional encourages and guides her so that the best choices can be made.
Effective care	The client is offered the most effective guidance and provision of care. Healthcare and guidance are professional, evidence-based, and free of personal values and normative beliefs.
Accessible care	The perinatal healthcare is available, accessible, and affordable.
Continuity of care	The client knows who is responsible for her care. The client experiences seamless transitions during transfers and when she enters and leaves the care system.
Information and education	The client has access to understandable information, tailored to her preferences and abilities.
Emotional support, empathy, and respect	The client feels heard and understood and receives psychosocial support where necessary.
Client-oriented environment	The client experiences an appropriate and pleasant environment.
Safety	The client experiences a safe environment. The client is noticeably prepared and informed of possible complications.
Transparency of care system	The client knows how the regional perinatal healthcare system works and which professionals work on what care level.
Transparency of cost	The client knows what she needs to pay regarding the provided healthcare.

^a^Translation by first author

Measuring clients’ experiences of perinatal healthcare poses a challenge, because there are different services (e.g. antenatal check-ups and screenings, care during childbirth, maternity care after childbirth, and youth healthcare), different healthcare providers (e.g. obstetricians, midwives, general practitioners, maternity assistants, and youth healthcare professionals), and time windows (e.g. the antepartum period, labour and childbirth, and the postpartum period) [8].

This is especially relevant in the Netherlands, where pregnant women might deal with even more healthcare providers due to the nation’s approach to risk selection [14]. Healthy clients with pregnancies and childbirths free of complications are guided by primary caregivers only, such as community midwives or general practitioners. Clients can choose whether they want to give birth at home or in the hospital. Clients who have pre-existing conditions or develop complications which can harm the mother and child, are transferred to the secondary or tertiary care level in the hospital. Secondary or tertiary healthcare providers such as clinical midwives or obstetricians will guide the clients throughout the pregnancy and childbirth. In the first week after childbirth, a community midwife together with a maternity care assistant will provide postpartum care. From the second week after childbirth, preventive child healthcare is provided by youth healthcare organisations to all new-borns. The approach to risk selection results in a high percentage of pregnant women, starting with an uncomplicated pregnancy guided by community midwives or general practitioners, being transferred when they develop complications and need specialist guidance of obstetricians or clinical midwives [15].

Since transfers are a notable characteristic of the perinatal healthcare system, researchers have tried to identify how they influence the satisfaction and experiences of clients [16–20]. It has been shown that transfers of care, especially during childbirth, have a negative influence on the satisfaction of clients [17, 18]. Even though factors have been identified that can explain the differences in satisfaction between women who were transferred or not, there is no list of quality criteria that clearly states what is essential for a satisfactory transfer during perinatal healthcare [18, 20, 21].

This paper addresses the lack of knowledge about aspects that determine quality of transfers in the chain of perinatal healthcare. We cannot assume that quality aspects regarding transfers are similar to quality aspects of the perinatal healthcare system in general (NPCF list), although there will be similar aspects. This paper therefore answers the following research question: How do clients experience transfers of care during pregnancy, childbirth, and the neonatal period, and how do these experiences compare to the established quality of care aspects the Dutch Patient Federation developed?

Investigating client experiences requires a qualitative approach, specifically one that can account for the richness and potential diversity of experiences. Over time, it became apparent to researchers that narratives could be used as a tool to understand experiences [22, 23] For example, narrative analyses have been used to provide insight into the broader context of illness experiences of people with neuromuscular diseases, to assess the role of patients in Dutch hospitals, and to explore how experiences of patients might contribute to improving quality of care [10, 24, 25].

## Method

### Study design

The data reported in this article are part of a narrative study of women who recently gave birth who were asked about their experiences of transfers between healthcare professionals during pregnancy, childbirth, or the neonatal period. The data were collected between April 2016 and August 2016.

### Population and setting

In 2015, 250 women living in the catchment area of one of the three Dutch Neonatal Intensive Care Units (NICUs) in the eastern part of the Netherlands participated in an observational, prospective, cohort study. The respondents were monitored from the moment the pregnancy was confirmed until the first month after delivery. During these 9 to 10 months, respondents received three questionnaires about their experiences of perinatal care. Of the 250 women, 44 were invited to participate in a successive qualitative research about their experiences of transfers of care. Respondents were selected if they – at any point during pregnancy, labour, childbirth or the neonatal period – experienced any of the following transfers between healthcare professionals: 1) guided by multiple community midwives during pregnancy, 2) transferred from the community midwife to an obstetrician or clinical midwife during pregnancy or childbirth, 3) transferred between multiple obstetricians or clinical midwives during childbirth, 4) transferred from the community midwife or obstetrician to the maternity assistant after childbirth, or 5) transferred from the maternity assistant to youth healthcare.

### Data collection

All 44 women received a letter in which the study was introduced and that explained why they were selected for participation in the qualitative research. The letter also mentioned that an incentive in the form of a gift coupon would be given to respondents who provided a story. The envelope contained an informed consent form and a return envelope, and instructions on how to write their experiences in the form of a narrative.

We wanted to collect experiences of clients with the least possible interference from researchers or other clients. Therefore, written narratives were chosen above performing interviews or focus groups. The use of such narratives reduces the risk of interview bias and the analysing takes less time than transcribing interviews or focus group recordings [26]. In order to ensure that the narratives were as detailed and complete as possible, respondents were given targeted instructions. First, they were asked to imagine that they were telling their story to a friend or wanted to share it on a forum for (expecting) mothers. We were interested in their personal experiences when they were transferred between healthcare providers. Second, the respondents were asked to give a broad overview of the events related to the transfer. Third, we indicated that we were interested in how they felt, what was important to them and what left an impression during these events. Fourth, participants were asked to remember the transfer they experienced during pregnancy, childbirth, or the neonatal period. From that position, they were asked to share their narrative about their experiences of the transfer. All respondents were given the choice to write their narrative on paper or on a computer, using a link and password that were given in the letter (see Additional file 1 for full instructions).

We received 17 narratives, of which nine were written on paper and eight were typed on a computer. The length of the narratives varied between 200 and 4000 words. All narratives were checked for and, if necessary, edited to guarantee anonymity.

### Analysis

Storyline analysis was used to identify story types that are indicative of various ways of giving meaning to a transfer of care [27]. Storyline analysis consists of three levels: story content and structure, interactional context of the story, and wider societal-cultural context. For the purpose of this article, only the first level was analysed. This method has previously been applied in the context of patient narratives and healthcare [25, 28, 29].

**Fig. 1:**
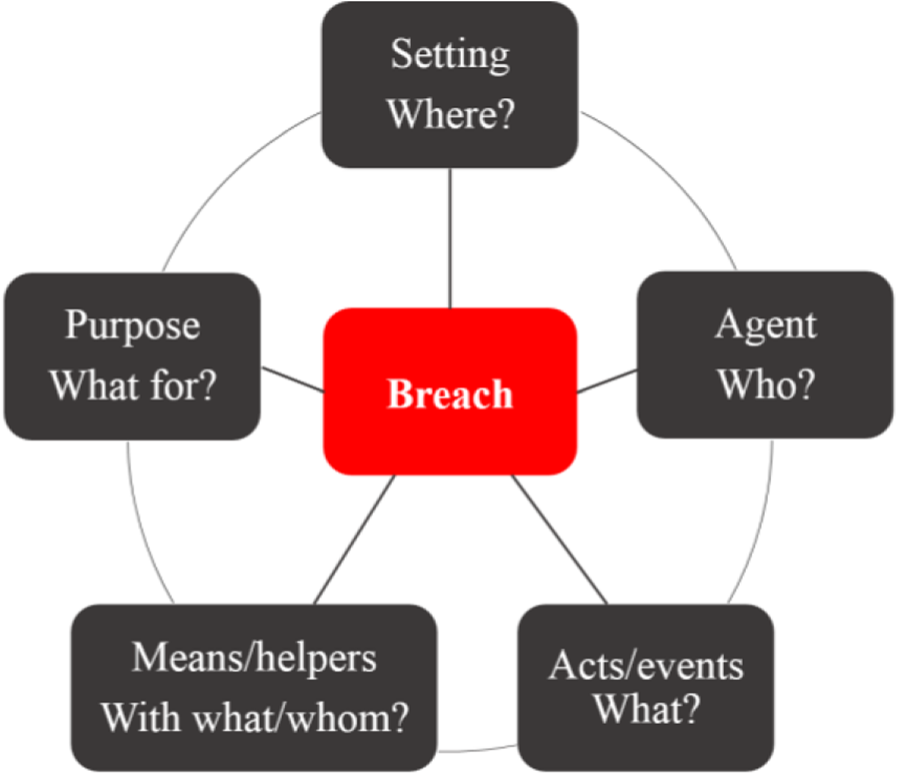
Pentad of storyline elements. Source: ©Anneke Sools, Storylab University of Twente (inspired by Burke, 1969 [33])

A storyline has five elements, connected as a coherent whole called a pentad (Fig. 1) [27]: (a) the agent – the main character of the story; (b) the setting or location – where the story takes place; (c) the acts and events – what the main characters do and what happens to the main character; (d) means and/or helpers – what or who is helpful for accomplishing the purpose; (e) the purpose – why the story develops, or a feared or desired goal. The storyline method assumes that there is an imbalance between two storyline elements, called the breach, in each story [30].

The story elements of each narrative were analysed and constructed into a storyline. This was repeated for all 17 narratives. Thereafter, the 17 storylines were compared with each other in order to find patterns across individual narratives. This was followed by comparing the identified story types with the quality aspects the NPCF developed. In this comparative process, quality aspects respondents mentioned in their narratives were labelled as similar, complementary or new when compared to the NPCF list of quality aspects.

To enhance reliability, the following measures were taken to increase intersubjective agreement. The identification of the storylines and story types were conducted by the first and second author and critically reviewed by the co-authors at various stages until agreement between researchers was reached. The third step was conducted by the first author and critically reviewed by all co-authors.

## Results

### Client experiences of transfers of care

In this first part of the results, we present four story types that were identified. They portray four distinct ways of how clients experienced a transfer during pregnancy, childbirth, or the neonatal period. The story types are derived from varying numbers of narratives: Type one is derived from nine client narratives, type two from four narratives, type three from three narratives and type four from one narrative.

### Type 1: Disconnected transfers of care lead to uncertainties

#### Main agent

Typically for this story type, the agent (protagonist) is pregnant for the first time and is not yet familiar with this situation: *“I was ignorant with my first pregnancy and did not really know what was going to happen”* (N1). Also characteristically for story type 1, is that the agent has a ‘medical’ pregnancy with multiple complications (e.g. high blood pressure, bleeding, or infections) instead of a natural pregnancy with an uncomplicated childbirth: *“There were minor problems that could have major implications for the baby all the time”* (N2)*.* As a consequence, she has a lot of contact moments with different healthcare providers.

The healthcare providers are often qualified differently, because they work at different levels of care and have different professions. The healthcare providers are mentioned hierarchically. The client seems to have more trust in the healthcare provider with the highest qualification and shows doubts and distrustful feelings towards the healthcare provider who is less qualified: *“It confused me that my trusted midwife reacted unconcerned about something that could be so dangerous according to the ‘higher’ educated obstetrician”* (N2)*.* The client feels insecure, due to the different and sometimes even opposing approaches and advices, and is unsure which approach and advice to follow: *“It is strange that the midwife gave such different information about this”* (N2)*.*

#### Setting

It is typical for this type of story that the setting includes multiple healthcare organisations. Most narratives of this type have in common that a transfer from the primary care level (e.g. a community midwife practice) to the secondary care level (e.g. a hospital) is inevitable due to complications. The setting is value loaded, and the client appreciates the hospital setting less than the community midwife practice. *“How I experienced the transfer? Detached, you no longer belong to the intimate midwife practice, you have to ‘swim’ in the large hospital where everyone is emotionally detached”* (N2)*.* In these narratives, the midwife’s practice is described as a cosy setting, where it is possible to have a connection with the healthcare provider. The hospital, on the other hand, is perceived negatively. It is described as a setting that confirms the client’s complications and ‘not-natural’ pregnancy and creates unpleasant surroundings: *“They were friendly at the hospital, but the hospital still leaves a bitter taste in the mouth”* (N3)*.*

#### Purpose

The client in this story type has a difficult pregnancy and childbirth due to complications and medical interventions. She expects that her healthcare providers (because of their expected professional expertise), support and guide her through the process of pregnancy and childbirth. She wants to stay involved in the whole process and have good contact with her healthcare providers:

*“Ever-changing healthcare providers guided me throughout the pregnancy and childbirth, I felt no connection or relationship with them and that is just what I need to feel safe during childbirth”* (N1)*.*

*“I wanted the follow-up appointment with the obstetrician who treated me, but even though I made an appointment with her, I met someone else. No feedback, no follow-up or closure. No more guidance”* (N1)*.*

The purpose of the client in this story type is to clearly indicate what she missed during the pregnancy or childbirth. These clients want to see that the healthcare providers are attainable and that there is a possibility to discuss concern.”

#### Acts and events

The client had no control over the dissimilar approaches and different types of advice. The different medical approaches and advice a client could encounter may lead to: 1) confusion due to the circumstances, in which the client does not know who is in control and what is happening: *“I was emotional because of the uncertainty”* (N4), 2) a loss of the client’s confidence and trust in healthcare providers, or 3) a feeling that the client is left alone, because the care is transferred often and there is no continuity of care: *“I felt terribly abandoned and poorly monitored”* (N5).

#### Means/helpers

When a client experiences a transfer between healthcare providers, she does not take on an active role. Afterwards, she often regrets not standing up for herself. The client develops an active attitude after the whole experience, which helps her to consider requesting an evaluative conversation with her healthcare provider. Evaluative conversations are used as a way to share experiences about *“what went well, what went less well and what should change next time”* (N6)*.* This active role is presented as a consequence of negative experiences rather than a desired goal from the start.

#### Breach

This type of story contains criticism about the poor communication, lack of involvement and support, resulting from transfers between multiple healthcare providers *“like a ping-pong ball”* (N3). As a result, the reasons behind the multiple transfers remain unknown to the client and she is stuck with a nagging feeling that her pregnancy and childbirth went by without her involvement: *“The worst thing was that I thought: ‘Okay, this is supposed to be my son, and that was only because my husband brought him to me and because it was a boy”* (N7)*.*

### Type 2: Seamless transfers of care due to proper collaboration lead to positive experiences

#### Main agent

In this type of story, the protagonist is most often an experienced mother. She has given birth before and is familiar with the perinatal healthcare system in the Netherlands. This becomes clear, among other matters, when the client explains what she likes or not. Her previous experience with perinatal healthcare forms the backdrop and creates the expectations for the current pregnancy, childbirth, or neonatal period. She becomes focused on negative past experiences and decides that she wants a more positive experience this time around: *“With the third pregnancy, I decided that matters had to change”* (N9)*.*

#### Setting

Narratives of this type take place in medical (e.g. hospital) as well as natural (e.g. home) settings. An overarching characteristic is that the client describes the setting as a place where she feels welcome and safe. The home of the client and midwife practices are mentioned as such settings. The client describes that she felt well looked after and was involved in the decision-making process.

#### Purpose

The client in this type of story fears a repeat of the past. The desired purpose of the client is to experience a smooth and natural perinatal period: *“I thought that it never ran smoothly (i.e. the last four pregnancies), and this time I wanted to feel completely relaxed in the week after giving birth”* (N8).

#### Acts/events

It is typical for this type of story that the client takes steps aimed at having a positive experience. For example, a client said: *“Therefore, I decided to switch maternity care organisations”* (N10)*.* Another client made an agreement about her treatment with her healthcare provider: *“I would stay under supervision of the obstetrician if the weight of the baby at 40 weeks was estimated to be more than 4 kg; if not, I could make the choice about who would guide me”* (N9)*.* These steps do not necessarily guarantee a more positive experience. However, the client in this story type takes the initiative to change something, to minimise the chance of repeating past negative experiences. She shows awareness of her ability to affect change in the situation and assumes an active role.

#### Means/helpers

In this type of story, the collaboration between the healthcare providers is described as seamless, natural and unnoticeable: *“The midwives and maternity care assistants did not know each other, but I did not notice it – they just seemed to be colleagues”* (N8)*.* In these narratives, the clients mentions two elements involved in a seamless transfer of care. First, the alignment between healthcare providers goes hand in hand with trusting each other’s expertise:

*“I thought it was nice to see that they were so well-aligned”* (R29);

*“The midwife trusted the expertise of the maternity care assistant entirely”* (N8)*;*

*“The midwife informed the hospital well and the hospital received and understood the communicated information.”*

Second, the alignment between clients and healthcare providers took place through consultation, dialogue, participation, and providing information:

*“We discussed everything and I liked that I was there myself, because it is about me”* (N8);

*“She told me all the different possibilities, one of which was a transfer to the hospital”* (N10).

#### Breach

The client learned a lesson from past experiences and decided that it would not happen again. The past experiences and her determination can lead to a satisfactory experience. The client adopted an active role and became involved in her healthcare process: *“I am very glad that I did this, because this time I was 100% satisfied with how it went”* (N8)*.* However, even with the efforts of the client, vulnerability remains as there is no assurance that the expectations can be met. Even after switching healthcare providers, it is possible that the situation will not improve.

### Type 3: Transfers of care lead to disruption of patient-provider connectedness

#### Main agent

In this story type, the main agent extends beyond the mother or pregnant woman and also includes family members (e.g. partner and/or child/children). This protagonist is different from the protagonists in the other story types, in which the narratives are mostly about the feelings of the client. Typical for this story type is that the impact of a transfer on the life and routine of the family members is described in these narratives.

#### Setting

Typically, the setting of this story is a place where the patient-provider connectedness is very important to the client. In this study, that setting is most often at home and associated concepts of relaxation and calmness are mentioned in these narratives.

#### Purpose

The client in this story type aims for a relaxed and carefree perinatal period: *“So all in all the days were less relaxed than I hoped”* (N12)*.* The family members and healthcare professionals understand each other and are aware of the preferences and expectations.

#### Acts/events

In some cases, a transfer is unavoidable (e.g. when a healthcare professional becomes ill). This type of story implies that the family receives a new healthcare provider and does not feel the same patient-provider connectedness after the transfer: *“The connection was missing completely”* (N13)*.* The family then blames the healthcare organisation for not cooperating with the family. For example, it is unclear who takes over the healthcare provision and it is often not possible for the family to choose a replacement: *“We really wanted to know who was coming and at what time”* (N12)*.* The transfer from a situation in which the family was positive and comfortable to a situation that was unclear and unwanted contributed to a negative experience: *“We would have liked it more if we could have ‘kept’ our first maternity care assistant”* (N13)*.*

#### Means/helpers

Typically, the client describes how the family (the mother and other family members) were positive and satisfied with the care prior to the transfer: *“This woman was excellent”* (N12)*.* A client said, *“It was so nice that we already had a connection, I felt safe with her right away”* (N12)*.* The healthcare provider knew what was expected of her/him and could work independently*: “She just did what was necessary; it made me feel comfortable that I knew she had it under control and I could let go”* (N12)*.*

The period before the transfer was very much as the client wanted and wished. In this type of story, the client was so satisfied with the prior healthcare provider that the new healthcare provider, per definition, could not measure up to that level. The client kept comparing the two healthcare providers with each other and was unsatisfied when the new healthcare provider did not measure up to the performance of the previous provider. The client tries to find a polite and socially acceptable way of expressing her needs without disrupting the relationship between her and the healthcare provider, in a way that takes into account her dependence on the healthcare provider: *“I told her that I did not like it, but that it did not go smoothly. I was probably too direct. Eventually, we worked it out”* (N12)*.*

#### Breach

In this type of story, the client is not satisfied after she experiences a transfer between healthcare providers. However, she decides not to request another healthcare provider: *“It seemed rude to ask for another [healthcare provider]”* (N13). This meant that she and her family stayed dissatisfied with the current situation and did not have the possibility of finding a suitable healthcare provider. The negative feelings are not directed to the healthcare provider personally. Often the healthcare provider is well-informed about the family and completes the tasks without problems: *“She was well-informed; that was not the problem”* (N13)*.* The problem is the missing connection between the family and the new healthcare provider. It also seems difficult to find the correct way of expressing your needs without disturbing the patient-provider connectedness. In this story type, raising the problem leads to unsatisfactory transfers. The manner of communication between the client and healthcare provider is an important part of the desired purpose.

### Type 4: Transfer of care is initiated by the client to make pregnancy and childbirth dreams come true

#### Main agent

Similar to story type 2, the protagonist in this type of story is a client who is typically experienced and has given birth multiple times. What is unique in this story is that she has a special wish or dream about what her healthcare provision should look like: *“With this fourth pregnancy, I wanted a water birth; a beautiful closure of four pregnancies”*(N14). This type of client does not want to steer away from her dream. The client is vigilant and has control over her own body.

#### Setting

In this story type, the client does not explicitly mention a physical setting, such as a hospital or home. In this story type the client only has eyes for the execution of her dream scenario, the physical setting goes unnoticed. This scenario is detailed and uncommon. In this situation, the setting (i.e. hospital, home) where the water birth takes place is not important to the client. The client makes sure that the involved healthcare providers are informed about her wishes and expectations.

#### Purpose

The purpose is explicit in this story type. The client envisions a specific scenario of how to give birth and does not want to deviate from it. She believes that the scenario is realistic and she will make sure that her dreams come true.

#### Acts/events

In this situation, the client stays in control. She acts immediately when she notices that her dream scenario is in jeopardy. For example, the client will specifically enquire if her scenario (e.g. water birth) is achievable: *“I asked specifically how it would go if an unqualified midwife (i.e. water birth) would be working during my delivery”* (N14). Second*,* the client seeks reassurance that her scenario is possible, and third, she is prepared to transfer to a different healthcare provider when her original healthcare providers do not satisfy her expectations. When it becomes apparent that the client’s dream scenario is not possible, she feels misunderstood and upset: *“That afternoon, I drove home in tears and immediately called three other midwife practices”* (N14)*.* These feelings were not present in the beginning of the story, but surface as a consequence of the unexpected events.

#### Means/helpers

It seems that not all healthcare providers have the qualification of guiding pregnant or childbearing women with their dream scenarios: *“If you guide water births, then all midwives of that practice should be qualified, otherwise the clients cannot count on them”* (N14)*.* A transfer between midwife practices is conceivable when a client has such specific wishes (e.g. water birth) and her initial midwife practice cannot satisfy her expectations.

#### Breach

In this story type, the transfer of care is an initiative of the client herself. In the beginning of this story type, the client believes and expects that the healthcare organisation can provide the service necessary to fulfil her dream delivery. In the course of the story, the client loses confidence and trust in the healthcare organisation and therefore decides to transfer.

### Comparison between quality aspects found in narratives and quality aspects from the NPCF list

In this second part of the results, we present how the transfer-related quality aspects as highlighted in the four story types compare to the NPCF list (Table 2). The table consists of three sections which are presented per story type. First, the quality aspects recognised in the narratives are presented. Second, the quality aspects from the NPCF list that match the aspects found in the narratives are shown. Third, we added if the quality aspects found in the narratives were similar, complementary or ‘new’ aspects when matched to the quality aspects from the NPCF list [13]. The label ‘complementary’ was given if the aspects were an addition of the quality aspects from the NPCF, and can be used to elaborate on the NPCF list.

**Table 2:** Quality aspects from the story types matched as similar/complementary/new to aspects from the NPCF list

Story type	Quality aspects recognised in the narratives	Matching quality aspects (NPCF list)	Similar/ complementary/new
1. Disconnected transfers of care lead to uncertainties	Evaluative conversation as closure;	Transparency of care system	Similar
	Involvement of client, even though multiple healthcare providers are involved with provision;	Autonomy	Similar
	Adapt advice and approaches, especially when different professions are involved.	Effective care	Similar
2. Seamless transfers of care due to proper collaboration lead to positive experiences	Previous experiences form expectations;	No matching aspect	New
	Comfortable and safe environment contributes to positive experience;	Client-oriented environment and safety	Complementary
	Mutual trust/alignment between healthcare providers;	Continuity of care	Complementary
	Explanation by healthcare providers about possible outcomes;	Autonomy and effective care	Similar
	Client is aware that she can change healthcare providers.	Autonomy	Similar
3. Transfers of care lead to disruption of patient-provider connectedness	Transfers have an influence on the family (partner and children);	Emotional support, empathy and respect	Complementary
	Patient-provider connectedness is important to clients, without this, the expectations of each other are unclear.	Emotional support, empathy and respect	Complementary
4. Transfer of care is initiated by the client to make pregnancy and childbirth dreams come true	Being able to offer a solution or being flexible as healthcare provider, especially when uncommon scenarios are desirable;	Effective care	Complementary
	Uncomplicated transfer between healthcare organisations;	Autonomy	Similar
	Information about the course of events should be made clear by healthcare providers.	Information and education	Similar

Eight out of ten quality aspects included in the NPCF list were mentioned in the narratives. ‘Accessible care’, and ‘Transparency of cost’ were the two quality aspects that were not represented in the story types. In story type one, the mentioned quality aspects were all constructed as being similar to those mentioned in the NPCF list. Quality aspects in story type one were mainly about the role of the client and shared decision-making.

In story type two, four quality aspects from the NPCF list were also mentioned in the narratives. A complementary quality aspect to the ‘Client-oriented environment’ and ‘Safety’ aspects was that clients who felt comfortable and safe in a care setting were more likely to have good experiences with a transfer of care. Optimal collaboration between healthcare providers during transfers of care is included in the NPCF list as being important for ‘Continuity of care.’ Adding to this, we found that clients have positive experiences when they notice that healthcare providers trust each other and align their care, especially when they have different professions. Particularly in story type two, previous experience with a pregnancy or childbirth influenced the expectations of the current pregnancy. Negative prior experiences created fear and concerns, whereas positive prior experiences created high expectations from the healthcare providers. This aspect was not included in any NPCF quality aspect, and was therefore labelled as a new quality aspect.

In story type three, quality aspects concerning patient-provider connectedness were mentioned in the narratives, which matched the quality aspect ‘Emotional support, empathy, and respect.’ In the narratives, we found aspects that a transfer can be intrusive for the entire family, not just the pregnant woman. When clients and healthcare providers feel connected, the communication about what is expected from each other also runs more smoothly. We found evidence in all four story types that clients find it pleasant when transfers between healthcare providers take place smoothly. They want it to be quick, seamless, and without too much involvement from their side, which matches the description of the quality aspect ‘Autonomy’ on the NPCF list. In the quality aspect ‘Effective care,’ it is mentioned that the healthcare provider is aware of the client’s expectations and wishes concerning pregnancy and childbirth and that deviations from these expectations and wishes must be discussed with the client [13].

In the narrative of story type four, it appears that this is especially true for more uncommon wishes, such as water births. Clients want clarity about whether their expectations can be satisfied. Otherwise they ask for a solution or flexible attitude of their healthcare providers. Furthermore, in this story type the NPCF list aspect 'Information and education' was mentioned. The information about the course of events surrounding a water birth were incomplete, which resulted in a disappointed client.

## Discussion

When research is targeted towards exploring clients’ experiences with perinatal healthcare, the researcher needs to be aware that those experiences are detailed and highly emotional. Hence, it is a challenge to explore the quality of care from the client’s perspective. This paper answers the research question: How do clients experience transfers of care during pregnancy, childbirth, and the neonatal period, and how do these experiences compare to the established quality of care aspects the Dutch Patient Federation developed?

Our analysis of transfer experiences during client’s perinatal period resulted in four story types: 1) Disconnected transfers of care lead to uncertainties; 2) Seamless transfers of care due to proper collaboration lead to positive experiences; 3) Transfers of care lead to disruption of patient-provider connectedness; 4) Transfer of care is initiated by the client to make pregnancy or childbirth dreams come true. Overall, these types show that both the level and valence of previous experiences of transfers impact the expectations of the current pregnancy and the consequent evaluation of the pregnancy.

After identifying the quality aspects derived from the narratives, we observed that most of these aspects are similar to those included on the NPCF list. Especially, experiences concerning the interaction between the client and the healthcare provider (‘Autonomy,’ ‘Effective care,’ ‘Safety,’ ‘Continuity of care,’ ‘Information and education’, and ‘Emotional support, empathy, and respect’) were mentioned as being very important to clients who were transferred. These results seem to be consistent with research that related negative effects of transfers to the feeling of not being in control and absent shared decision-making [17, 31].

Aspects concerning the organisational structure of healthcare settings (‘Accessible care,’ ‘Client-oriented environment,’ and ‘Transparency of care and cost’) were hardly mentioned in the narratives. It is possible that these aspects satisfied the expectations of the clients, so that they did not feel the need to mention them in their narratives. Also, medical costs during pregnancy and childbirth seem to be insignificant to our respondents. The reason could be that medical costs are not discussed during a transfer of care or that the bulk of perinatal care is included in the mandatory basic health insurance [32]. Based on our results we should suggest removing the quality aspect “Transparency of care and cost” as an influencing factor on the experiences of clients with transfers of care.

The newly found quality aspect shows that prior experiences with transfers of care influence the current pregnancy or childbirth. Clients had positive experiences when they previously experienced transfers between healthcare providers (type two and four). This was the case for clients with an initial negative experience as well as for clients with an initial positive experience. Both types of clients knew what to expect and could therefore make changes or adjustments in their care process. In our study, the course of the pregnancy influences the experiences with the transfer between healthcare providers. Clients who had a complicated pregnancy (type one) with multiple health issues often wrote a negative story about their experiences with transfers. They experienced a fearful period, with lots of medical interventions. Healthcare providers should be more aware of clients’ prior experiences with perinatal healthcare, either positive or negative, so that they can adapt their care provision accordingly. Those experiences can be discussed during the first check-up with the midwife or obstetrician or even be written down, such as with this study. The transparency of fears, concerns, wishes, and dreams gives healthcare providers the opportunity to improve their role in assisting women achieve their expectations.

Client experiences in the form of narratives give us the opportunity to not only identify aspects of quality, but to also consider which quality aspects are more important to specific types of clients in particular settings. For example, clients barely described their experiences with the actual transfer journey (transport from a home setting to the hospital), presumably because they accepted that the transfer was a medical necessity. However, they did describe surrounding events that caused the most impact, such as losing the connection with the previous healthcare provider (type three), experiencing different types of advice and approaches from the healthcare providers, or having the possibility to talk with a midwife or obstetrician about their experiences (type one). Preparing clients for the multiple potential courses of pregnancy and childbirth is recommended. This is especially true for clients who are pregnant for the first time and encounter complications. Due to these complications, multiple transfers are unavoidable. Guiding the clients through these changes can positively influence their experiences. In the narratives, the clients mentioned that due to changing healthcare providers, types of advice, and approaches they would value a case manager being assigned to them. The case manager is responsible for ensuring that the care is client-centred and can provide support, expertise, and evaluate individual needs. Assigning case managers to inexperienced pregnant women with complications can enhance the quality of care and the overall experience.

To our knowledge this is the first study which uses written narratives to analyse clients’ experiences with transfers in the perinatal healthcare. By giving clients the opportunity to write down what was most important to them in great detail, without the possible interference from researchers, we were provided with rich accounts of pregnancy and childbirth experiences. However, we kept the amount of instructions to a bare minimum, which means there is a possibility that stories came out less valuable for the purpose of this study. Another limitation of our study is that the story types and quality aspects were all constructed from Dutch narratives. For future research, it would be interesting to repeat this research with clients from other perinatal care systems, and do an international comparison. Such a comparison can give insight into which story types and quality aspects are unique to the Dutch context and which are also valid in a broader context.

## Conclusion

This study unveiled four story types about how clients experienced transfers of care. Transfers of care during pregnancy and childbirth affect clients greatly and influence their experiences with perinatal healthcare. Vice versa, previous experiences influence how clients proceed and expect the next pregnancy, childbirth, and possible transfers of care. Quality aspects highlighted in the narratives compare well with the quality aspects the NPCF developed. However, identifying story types provides a much richer insight into what clients think is important, what caused the most impact and what they want to see differently. Healthcare providers could use the story types to determine certain types of clients and situations and better prepare them for potential transfers. Good communication, seamless transfers, and maintaining clients’ autonomy can all contribute to improving the transfer process for clients.

## Additional file

Additional file 1: Experiences with transfers of care during pregnancy, childbirth, or the neonatal period. Targeted instructions for writing a narrative. (DOCX 14 kb)

## Abbreviations

NICU: Neonatale Intensive Care Unit (Neonatal Intensive Care Unit)
NPCF: Nederlandse Patiënten Consumenten Federatie (Dutch Patient Consumers Federation)
