# Supplementary material for: Through the client’s eyes: using narratives to explore experiences of care transfers during pregnancy, childbirth, and the neonatal period
Source: BMC Pregnancy Childbirth. 2017 Jun 12;17:182. doi: 10.1186/s12884-017-1369-6 (PMC5467260; doi:10.1186/s12884-017-1369-6)
Supplement: Additional file 1: — Experiences with transfers of care during pregnancy, childbirth, or the neonatal period. Targeted instructions for writing a narrative. (DOCX 14 kb) [file 12884_2017_1369_MOESM1_ESM.docx]

Additional file 1

**Experiences with transfers of care during pregnancy, childbirth, or the neonatal period**

Thank you for your interest in sharing your experience with transfers during perinatal healthcare. By writing down your experience and submitting it to be analysed in our study, you can make an important contribution to improving perinatal healthcare. To thank you for writing your story, you will receive a gift coupon worth 15 euros.

Instructions

Our request is that you describe your experience in the form of a story. Imagine that you are telling your own story to a friend or that you want to share your story on a forum for (expecting) women. We are interested in your personal experience when you were transferred from one care provider to another. The specific transfer we are referring to was disclosed in the letter we sent to your home address, together with the informed consent form.

We would like to know how you experienced the transfer, which aspects you thought were important, and the impressions you had at the time. Please do not only describe the events that took place, but also explain the effect on you. Describe the kind of situation you found yourself in at that time, the thoughts and feelings you had when the transfer took place, and how you feel right now. Please create your own story and try to not only recite medical events.

If you do not have the letter with the specific information about your transfer, or if you have questions regarding the study, please contact the researchers at the details of the letterhead.

Good luck with writing your story. We look forward to receiving it!

Sincerely, on behalf of the research team at Pregnancy and Birth Overijssel
